# Bioabsorbable vs. titanium screws in first metatarsophalangeal joint arthrodesis: a pilot randomized controlled trial with 2-year follow-up

**DOI:** 10.1007/s00402-026-06501-2

**Published:** 2026-09-09

**Authors:** Nikke Partio, Ville Mattila, Ville Waris, Heikki Mäenpää

**Affiliations:** 1 Tampere University Hospital, Tampere, Finland; 2 Mehiläinen Hospital, Mikkeli, Finland

**Keywords:** Metatarsophalangeal joint, Arthrodesis, Bioabsorbable implant, Randomized controlled trial

## Abstract

**Introduction:**

To assess the feasibility and preliminary clinical outcomes of bioabsorbable screw fixation in first metatarsophalangeal joint arthrodesis compared to conventional titanium screw fixation. Methods: In this prospective pilot randomized controlled trial, 30 patients were allocated 1:1 to bioabsorbable or titanium cannulated lag screw fixation. Arthrodesis was performed using a cup-and-cone technique at two Finnish hospitals. Full weight-bearing in an orthopaedic shoe was allowed after six weeks. Outcomes included AOFAS and VA-FAS FA-VAS scores, union rates, and complications assessed at 3, 6, 12, and 24 months.

**Results:**

Both groups showed significant functional improvement with no between-group differences. At 24 months, mean AOFAS scores were 89.1 (SD 2.7) in the titanium group and 90.0 (SD 0.0) in the bioabsorbable group (p = 0.23). Mean FA-VAS total scores (sum of pain, function, and complaints subscales) were 1948 (SE 59.4) in the titanium group and 1914 (SE 61.4) in the bioabsorbable group (p = 0.655). Union rate was 90% overall; nonunion occurred in one titanium and two bioabsorbable patients, all requiring revision. No implant-related complications or hardware removal surgeries were recorded. Degradation of bioabsorbable screws caused no adverse clinical or radiographic reactions.

**Conclusions:**

Bioabsorbable screws appear safe and feasible in first metatarsophalangeal joint arthrodesis, with preliminary outcomes comparable to titanium fixation. These findings support the conduct of a larger confirmatory trial.

**Trial registration:**

ClinicalTrials.gov, NCT03133039, registered 28 April 2017. https://clinicaltrials.gov/ct2/show/NCT03133039

## Introduction

Arthrodesis of the first metatarsophalangeal joint is a well-established and commonly performed surgical procedure for the treatment of arthritic first metatarsophalangeal joints with hallux valgus [1]. Several surgical techniques for first metatarsal phalangeal joint arthrodesis have been described [2–4]. Despite its frequent use, reported union rates over the past 30 years range from 56% to 100%, depending on technique [5, 6]. Currently, the “cup and cone” technique using dome-shaped reamers is recommended over flat cuts, as it allows for greater adjustability and facilitates optimal alignment of the hallux with similar complication rate [3, 7, 8].

The most common fixation methods include two crossing interfragmentary screws—typically titanium or stainless steel—and either a locking plate or a plate combined with an interfragmentary compression screw (IFCS) [3]. Recent systematic review demonstrates that dorsal plating with compression screws is the most stable fixation construct [9]. When considering screw fixation alone, intramedullary and fully threaded screws may offer superior strength and stability compared to cancellous interfragmentary screws [3]. Although titanium and stainless-steel hardware provide superior mechanical strength, implant prominence and hardware-related pain remain common concerns. Implant removal rate is estimated to be 8,5%, ranging from 0 to 17% after first metatarsal phalangeal joint arthrodesis [6, 10, 11]. Local discomfort due to the fixation material is the main cause for removal operation, but it is difficult to determine whether a certain type of hardware increases the risk of surgical revision for isolated discomfort.

Bioabsorbable implants are a well-established alternative to titanium for internal fixation of certain fractures and osteotomies [12]. However, only one study has investigated the use of bioabsorbable screws in first metatarsal phalangeal joint arthrodesis, with good results and without adverse reactions [13].

Although some recent studies have assessed results of arthrodesis procedure with bioabsorbable screws, there remains limited evidence regarding the use of bioabsorbable screws in first metatarsal phalangeal joint arthrodesis. The aim of this pilot randomized controlled trial was to assess the feasibility, safety, and preliminary clinical outcomes of bioabsorbable screw fixation in first metatarsophalangeal joint arthrodesis, using conventional titanium cannulated lag screws as the comparator, and to inform the design of a future larger-scale trial.

## Methods

### Study design and ethics

This study was a prospective, pilot randomized, parallel-group clinical trial comparing bioabsorbable and titanium screw fixation in first metatarsophalangeal joint arthrodesis. Patient recruitment was conducted between February 2015 and April 2023 at two Finnish hospitals: Mikkeli Central Hospital and Tampere University Hospital by principal investigator NP.

The study protocol was approved by the Regional Ethics Committee of Kuopio University Hospital, Finland (ETL code R10127). Local ethics committees and hospital districts granted institutional research permissions prior to study initiation. The study was conducted in accordance with the Declaration of Helsinki. All participants provided written informed consent before enrollment. Independent steering and monitoring committees oversaw the conduct of the trial. The study was registered at ClinicalTrials.gov (Identifier: NCT03133039, registered on 28 April 2017) and is reported in accordance with the CONSORT guidelines.

The protocol was signed by the principal investigator (NP) at each study site, and the investigator site file was maintained locally in accordance with good clinical practice. The authors confirm the accuracy of the data and analyses and adherence to the study protocol.

### Patients

Between February 2015 and April 2023, a total of 30 patients undergoing first metatarsophalangeal joint arthrodesis were enrolled and randomly assigned to one of two treatment groups: bioabsorbable screw fixation (*n *= 15) or titanium screw fixation (*n* = 15). Baseline demographic and clinical characteristics are presented in Table 1.

**Table 1:** Baseline demographic and clinical characteristics

Variable	Titanium screw (*n* = 15)	Bioabsorbable screw (*n* = 15)
Age (years)	58.2 ± 5.1	59.1 ± 4.6
BMI (kg/m²)	26.3 ± 2.6	28.9 ± 4.9
AOFAS score	63.5 ± 10.6	50.5 ± 17.7
FA-VAS total	1085.1 ± 221.0	954.3 ± 250.9
Sex (Male/Female)	4/11	5/10
Comorbidities*	Hypertension (*n* = 5) Hypercholesterolemia (*n* = 3)	Hypertension (*n* = 4) Hypercholesterolemia (*n* = 2)

*Comorbidities listed without statistical comparison

Eligible patients were adults aged 18 to 60 years with symptomatic first metatarsophalangeal joint pathology (arthritic hallux valgus) requiring arthrodesis after failure of nonoperative treatment, classified as Grade 3–4 according to the Coughlin-Shurnas classification [14]. Exclusion criteria included medically treated diabetes mellitus, rheumatoid arthritis, active smoking, traumatic etiology, previous foot surgery, congenital foot deformities, pregnancy, significant peripheral vascular disease, and inability to comply with postoperative care (e.g. alcohol abuse).

### Randomization and blinding

Eligible patients were randomly assigned in a 1:1 ratio to undergo operative treatment with a bioabsorbable screw or traditional titanium screw treatment. After enrollment, patients underwent randomization. Sealed envelopes were used for the randomization.

Once the indication for surgery had been established, all eligible patients were informed about the different surgical fixation options during a preoperative visit. Written informed consent was obtained before randomization.

Randomization was performed by the principal investigator (NP) using a 1:1 allocation ratio. Sealed opaque envelopes were used to allocate patients to either bioabsorbable or titanium screw fixation. Due to the nature of the implants, neither the surgeon nor the patient was blinded to the allocated treatment. However, patients were not explicitly informed of which screw type they had received until after their first postoperative follow-up visit, in order to minimize potential bias in patient-reported outcomes such as pain and satisfaction during the early recovery period.

### Bioabsorbable implant

Self-reinforced amorphous poly(lactic-co-glycolic) acid (PLGA) (85:15) offers a promising solution for bioresorbable bone fixation. Compared to more rapidly degrading PLGA compositions (e.g. 50:50), the higher lactide content slows hydrolysis due to reduced water uptake [15]. The amorphous structure ensures uniform degradation without residual crystalline regions, enabling predictable resorption. Self-reinforcement, achieved by orienting polymer chains into fibrils, significantly enhances mechanical properties like strength, stiffness, and elongation, without altering chemical composition. Clinically, SR-PLGA 85:15 maintains fixation strength for up to 12 weeks, with complete resorption typically occurring within 2–4 years [12, 16, 17].

### Surgical technique and postoperative care

**Fig. 1:**
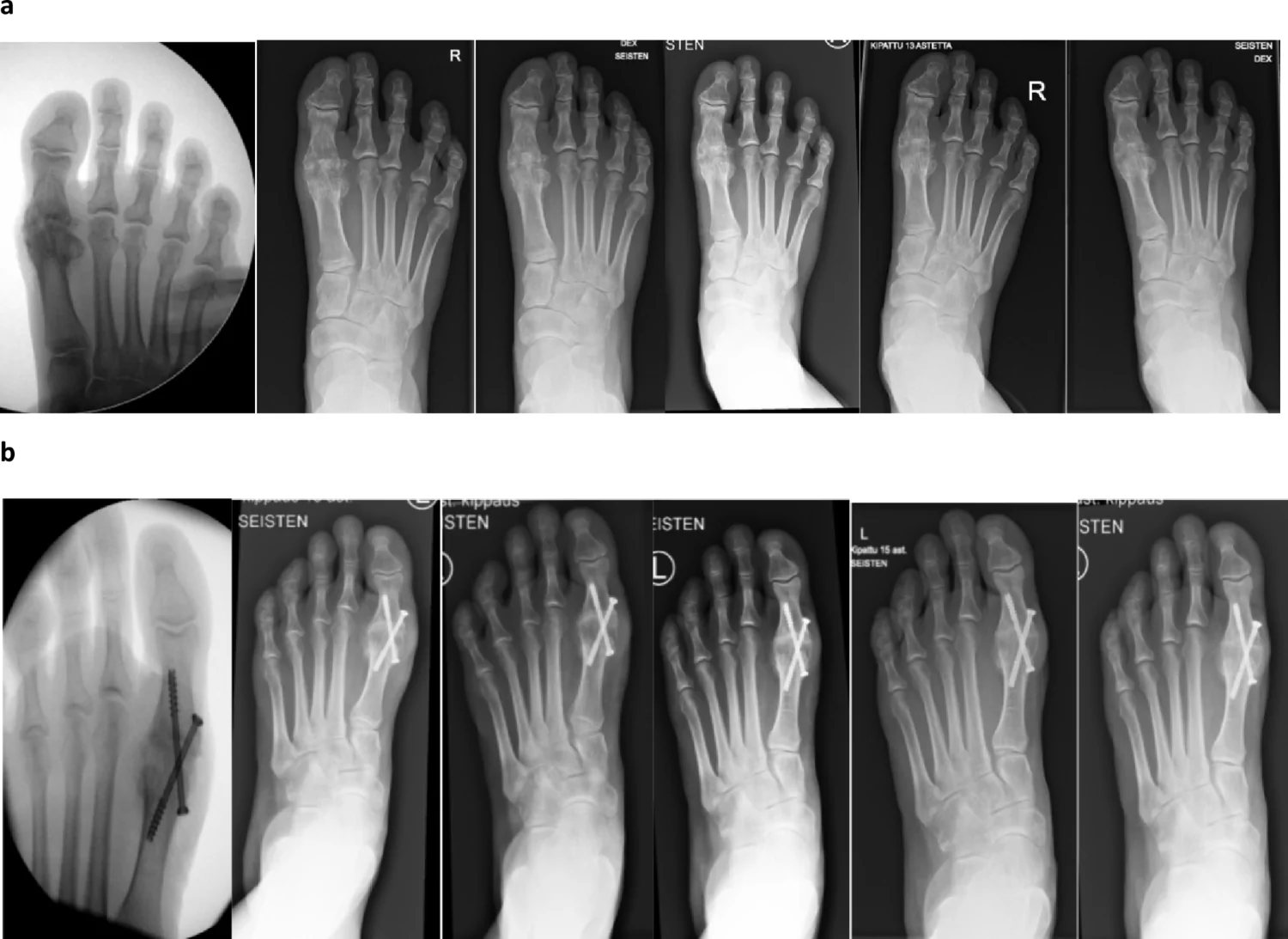
**a** Bioabsorbable screw fixation. Intraoperative image and follow-up radiographs at 6 weeks, 3 months, 6 months, 12 months, and 24 months. **b** Titanium screw fixation. Intraoperative image and follow-up radiographs at 6 weeks, 3 months, 6 months, 12 months, and 24 months

All surgeries were performed by the principal investigator (NP). Spinal anesthesia was employed. Cefuroxime 3 g iv. was given as prophylactic antibiotic, and tourniquet was used in tight at 280mmHg. Incision was made dorsomedially and capsule was opened with a straight incision. Joint resection was made with joint reamers. Two cannulated 4.0 mm bioabsorbable screws and two titanium alloy screws were used (bioabsorbable screws: Activa screw, Bioretec Ltd, Finland; titanium screws: Asnis screw, Stryker (Stryker Trauma GmbH/Stryker GmbH & Co. KG). In bioabsorbable group the first metatarsal phalangeal joint fusion was temporarily fixed with K-wires. The guide wire was then over drilled with a cannulated 2.7 mm drill and the channels were tapped without fully closing the osteotomy site. The two cannulated cortical lag screw was inserted dorsal-to-plantar over the osteotomy with K-wires. The first screw was inserted distal-to-proximal and the second screw proximal-to-distal over the arthrodesis site and second one distal-to-proximal over the arthrodesis site to opposite cortex (Fig. 1). The K-wires were then removed. Intraoperative X-ray was performed.

In the titanium screw group, the surgical technique and screw configuration were otherwise identical; however, the drilling was performed only through the near (anterior) cortex, and no tapping of the drilled channels was performed prior to screw insertion.

Full weight-bearing and active mobilization were allowed right away after the surgery. Stitches were removed after two weeks by nurse. Foot orthosis was used for six weeks. First outpatient visit with X-rays was performed at six weeks. The following outpatient visits with X-rays, AOFAS scoring and FA-VAS scoring were at 3, 6, 12 and 24 months.

### Outcome measures

The primary outcome was AOFAS measured at 2-year follow-up. Secondary outcomes included FA-VAS [18], union rate and hardware removal surgery. Complications like infection, nerve damage, bleeding and, malunion were also recorded. The data were collected during the research visits at 3, 6, 12 and 24 months.

The baseline and follow-up data was stored in paper portfolios that were analyzed after the last patient’s 2-year follow-up visit. The portfolios included the patients’ baseline characteristics, patient questionnaires, validated questionnaires, and case report files that contained information about group allocation.

### Statistical analysis

This study was designed as a pilot randomized controlled trial to evaluate the feasibility, safety, and preliminary clinical outcomes of bioabsorbable screws in first metatarsal phalangeal joint arthrodesis. As such, the primary aim was not to detect statistically significant differences between treatment groups, but rather to assess the viability of conducting a larger-scale trial and to gather initial data on clinical performance. So, the sample size was considered sufficient to evaluate recruitment capability, protocol adherence, and preliminary clinical outcomes.

Sample size calculations were performed using OpenEpi (Version 2.3) for a two-sample mean comparison of AOFAS scores. A clinically meaningful difference was defined as 23 points (SD 16), with an alpha level of 0.05 and power of 0.8. These parameters yielded a minimum sample size of 18 patients (9 per group). To account for potential dropouts, the recruitment target was set at 30 patients (15 per group).

## Results

### Primary outcome

**Fig. 2:**
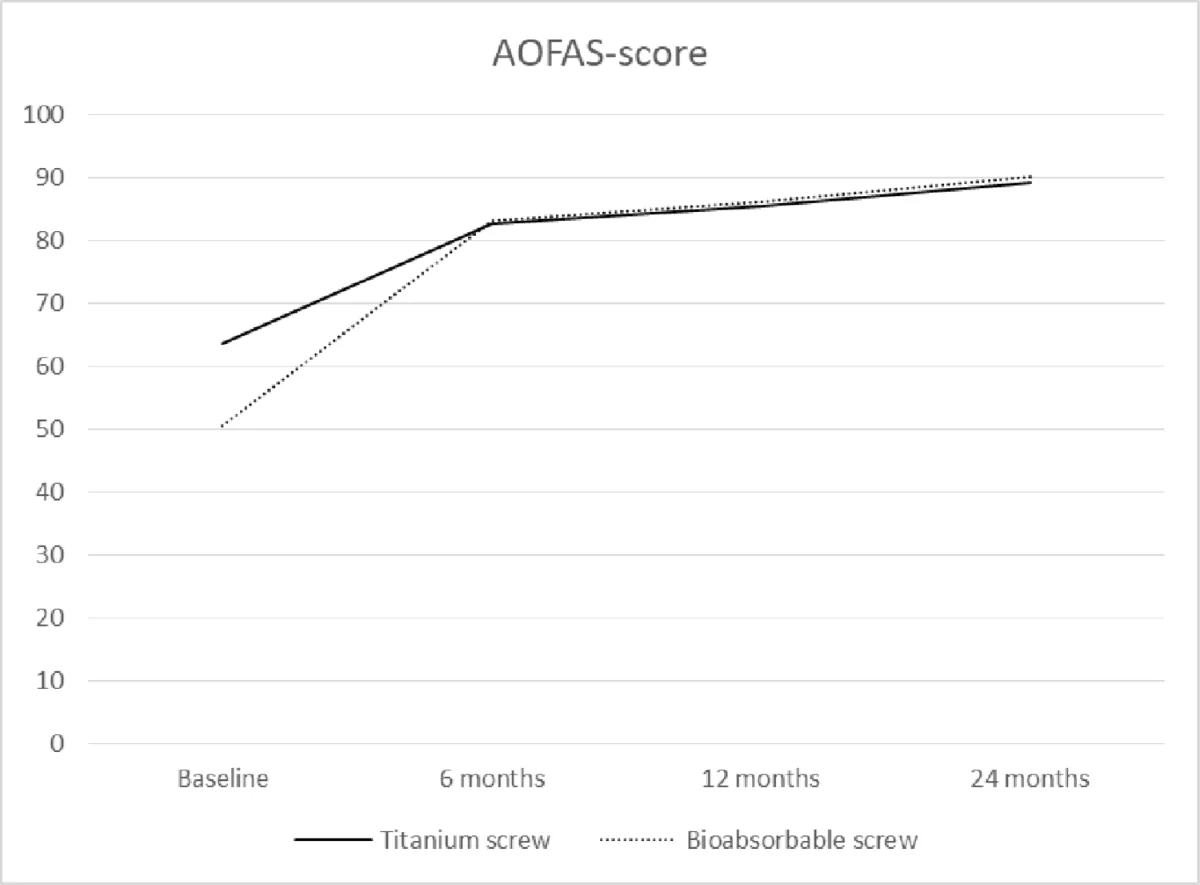
AOFAS score (mean)

At the 24-month follow-up, patients who had not undergone revision surgery were included in the analysis (titanium group *n* = 14, bioabsorbable group *n* = 13). The mean AOFAS score was 89.1 points (SD 2.7) in the titanium screw group and 90.0 points (SD 0.0) in the bioabsorbable screw group. The mean between-group difference was −0.9 points (95% CI −2.4 to 0.6; *p* = 0.23), indicating no statistically or clinically significant difference in functional outcome between the two fixation methods at 2 years (Fig. 2).

### Secondary outcomes

**Fig. 3:**
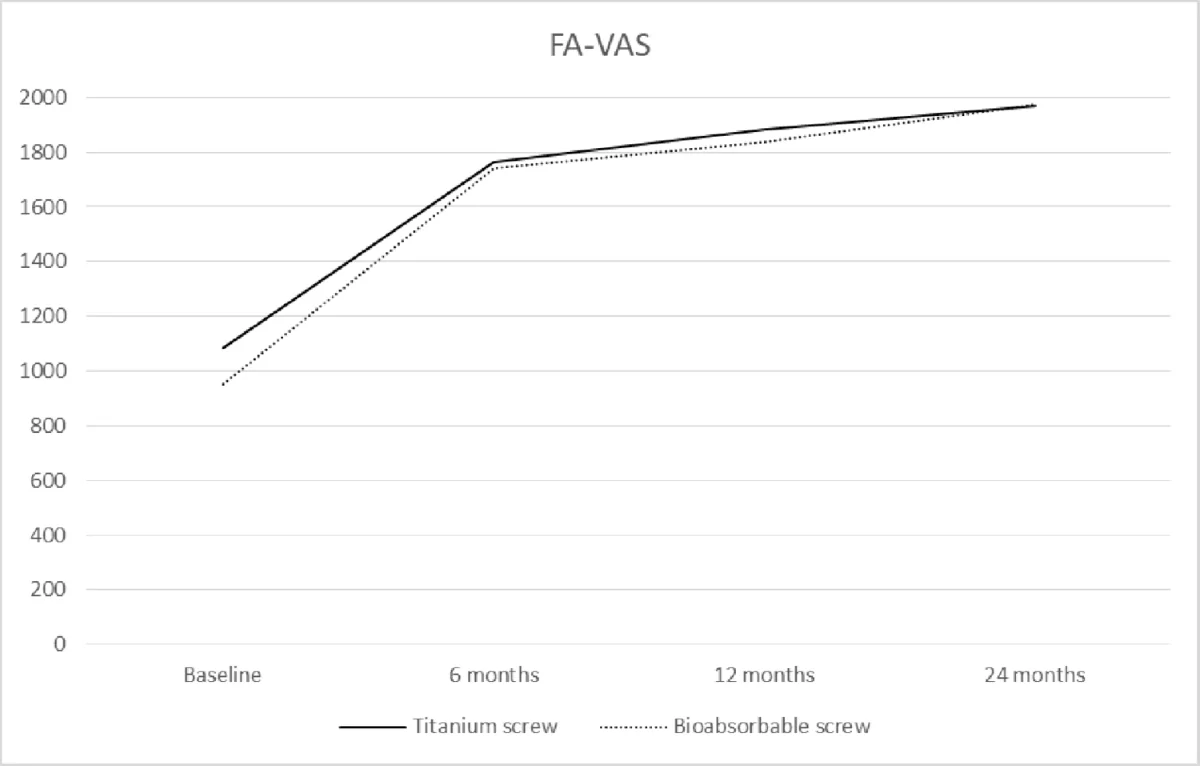
FA-VAS score (mean)

At the 24-month follow-up, patients who had not undergone revision surgery were included in the analysis (titanium group *n* = 14, bioabsorbable group *n* = 13). The mean FA-VAS total score (sum of pain, function, and complaints subscales) was 1948 points (SE 59.4) in the titanium screw group and 1914 points (SE 61.4) in the bioabsorbable screw group. The mean between-group difference was 34.3 points (95% CI −186.2 to 117.6; *p* = 0.655), indicating no statistically or clinically significant difference in functional outcome between the two fixation methods at 2 years (Fig. 3; Table 2).

There were no clinically significant differences in nonunion rates between the groups, with two cases in the bioabsorbable screw group and one in the titanium screw group. Preoperatively, the hallux valgus angle (HVA) was 25.8° in the titanium group and 27.0° in the bioabsorbable group. At the 2-year follow-up, the HVA had decreased to 11.1° in the titanium group and 10.9° in the bioabsorbable group.

The intermetatarsal angle (IMA) was 14.5° preoperatively in the titanium group and 12.3° in the bioabsorbable group. At 2 years, the IMA was 6.8° in the titanium group and 5.7° in the bioabsorbable group.

**Table 2:** FA-VAS score subgroups

	FA-VAS pain (mean ± SD)	FA-VAS function (mean ± SD)	FA-VAS complaints (mean ± SD)
Titanium			
Baseline	152.9 ± 53.2	665.7 ± 154.6	266.5 ± 67.3
6 month	370.5 ± 45.7	980.0 ± 88.0	412.0 ± 71.9
12 month	384.3 ± 45.6	1031.8 ± 114.0	466.4 ± 68.6
24 month	397.4 ± 6.0	1078.8 ± 27.9	494.0 ± 13.3
Bioabsorbable			
Baseline	133.5 ± 42.9	577.4 ± 154.2	243.5 ± 74.1
6 month	350.0 ± 79.3	976.5 ± 116.2	414.3 ± 84.5
12 month	377.6 ± 42.1	1024.4 ± 110.1	434.1 ± 121.6
24 month	399.8 ± 0.6	1078.3 ± 36.6	496.9 ± 4.5

### Adverse reactions

At the 2-year follow-up, one complication (1/15, 6.7%) was observed in the titanium group (nonunion). In the bioabsorbable group, there were two cases of nonunion (2/15, 13%). All three patients (3/30, 10%) required revision surgery due to nonunion. No implant-related complications like hardware removal or deep infections were reported. Additionally, four patients (13.3%) —two from the titanium group and two from the bioabsorbable group—experienced superficial infections, which were treated with oral antibiotics. There were not any adverse reactions during the operations, bioabsorbable screws did not break and arthrodesis line was well fitted in all cases. Postoperatively, no malunion or problems with pain or walking were observed, apart from the three nonunions. Degradation of the bioabsorbable screws did not cause any clinical or radiographical adverse reactions.

## Discussion

Our findings indicate that first metatarsal phalangeal joint arthrodesis with bioabsorbable screws could be a viable alternative fixation method for well selected patients. Additionally, the HVA and IMA were significantly reduced in all patients and did not change over time. At the two-year follow-up, the results were similar between the study groups.

In our study, there was no evidence of harmful tissue reactions or impaired healing like a foreign body reaction, painful erythematous papule or implant tracks [19, 20]. Bioabsorbable screws provide temporary support during tissue healing and gradually resorb, leaving no foreign material and restoring the original load-bearing capacity of the bone.

Despite good long-term results with first metatarsal phalangeal joint fusion, some patients report residual symptoms, such as plantar-medial pain, sesamoid pressure, and hardware irritation [5, 10]. We observed none of these in our study, and the overall results were good in both groups. Commonly reported complications include non-union, malposition, hardware removal, interphalangeal or metatarsocuneiform arthritis, metatarsalgia, and first-ray shortening [11]. Rashid et al. reported complication rates of 26% with plate and lag screw fixation, and 20% with a lag screw or plate alone [21]. A systematic review reported a complication rate of 23.1%, most commonly nonunion, delayed union, or implant removal [22]. In our series, the complication rate was 10% (3/30 nonunions: two in the bioabsorbable group and one in the titanium group), and there were no other complications. Importantly, no implant removal surgeries were required, whereas previous reports estimate removal rates at 8.5% [6, 10, 11].

Studies also suggest that bioabsorbable implants reduce the risk of infection, stress shielding, and peri-implant osteoporosis [22, 23]. Thus, these implants could help avoid secondary surgeries. The key question, however, is whether bioabsorbable screws can achieve a non-union rate comparable to titanium screws. Our study shows promising results that it is possible to achieve good union rate, without complications with include titanium alloy implants. Bioabsorbable fixation is initially rigid, but as it degrades, it is replaced by the patient’s own bone, and its initial strength is similar to cortical bone [24].

The limitations of our study include the small sample size and the fact that all procedures were performed by a single surgeon. This study was designed as a pilot trial; its primary aim was not to determine superiority between screw types, but to assess whether bioabsorbable screw fixation is feasible and safe for this indication, and the sample size was calculated a priori accordingly. The long recruitment period (February 2015 to April 2023) reflects the fact that all procedures were performed by a single surgeon and the strict, narrowly defined eligibility criteria, which limited the pool of suitable candidates. While bioabsorbable screws may offer benefits, these could not be conclusively demonstrated in our study. Nevertheless, our results are consistent with previous studies, showing no differences in complication rates. Although baseline FA-VAS and AOFAS scores were slightly worse in the bioabsorbable group, this did not influence the outcomes.

## Conclusion

Bioabsorbable screws are a good option for carefully selected patients undergoing first metatarsal phalangeal joint arthrodesis. This study shows that bioabsorbable screws perform similarly to titanium screws. Our study does not demonstrate superiority of either material but supports the use of both as acceptable fixation methods for first metatarsal phalangeal joint arthrodesis.

## Data Availability

The datasets generated and analysed during the current study are not publicly available due to patient privacy considerations but are available from the corresponding author on reasonable request.

## Abbreviations

AOFAS: American orthopaedic foot and ankle society score
BMI: Body mass index
CI: Confidence interval
CONSORT: Consolidated standards of reporting trials
FA-VAS: Foot and ankle visual analogue scale
HVA: Hallux valgus angle
IFCS: Interfragmentary compression screw
IMA: Intermetatarsal angle
iv: Intravenous
PLGA: Poly(lactic-co-glycolic) acid
RCT: Randomized controlled trial
SD: Standard deviation
SE: Standard error
SR-PLGA: Self-reinforced poly(lactic-co-glycolic) acid
